# New fossil species of ommatids (Coleoptera: Archostemata) from the Middle Mesozoic of China illuminating the phylogeny of Ommatidae

**DOI:** 10.1186/1471-2148-12-113

**Published:** 2012-07-09

**Authors:** Jingjing Tan, Yongjie Wang, Dong Ren, Xingke Yang

**Affiliations:** 1 Key Laboratory of Zoological Systematics and Evolution, Institute of Zoology, Chinese Academy of Sciences, Box 92, No. 1, Beichen West Road, Chaoyang District, Beijing 100101, People’s Republic of China; 2 College of Life Sciences, Capital Normal University, Beijing 100048, People’s Republic of China

**Keywords:** Ommatidae, *Omma*, *Tetraphalerus*, New species, Mesozoic, China

## Abstract

**Background:**

Ommatidae is arguably the “most ancestral” extant beetle family. Recent species of this group are only found in South America and Australia, but the fossil record reveals a much broader geographical distribution in the Mesozoic. Up to now, thirteen fossil genera with more than 100 species of ommatids have been described. However, the systematic relationships of the extant and extinct Ommatidae have remained obscure. Three constraint topologies were designed based on Kirejtshuk’s hypothesis, enforced the monophyly of *Tetraphalerus* + *Odontomma*, *Pareuryomma* + *Notocupes* and both respectively.

**Results:**

In this study, four new species, *Pareuryomma ancistrodonta* sp. nov., *Pareuryomma cardiobasis* sp. nov., *Omma delicata* sp. nov., and *Tetraphalerus decorosus* sp. nov., are described. Based on well-preserved fossil specimens and previously published data the phylogenetic relationships of extant and extinct lineages of Ommatidae were analyzed for the first time cladistically. Based on the results we propose a new classification with six tribes of Ommatidae: Pronotocupedini, Notocupedini, Lithocupedini, Brochocoleini, Ommatini and Tetraphalerini. These taxa replace the traditional four subfamilies.

**Conclusion:**

There is good support for the monophyly of the ingroup. Notocupedini, as defined by Ponomarenko, are paraphyletic. *Notocupoides* + *Eurydictyon* are the sister group of the remaining fossil and extant ommatids. Together they form the clade Pronotocupedini. Notocupedini and Lithocupedini are the next two branches. The tribe Brochocoleini is the sister group of a clade comprising Tetraphalerini and Ommatini.

## Background

Ommatidae is one of 4 or 5 extant beetle families of the suborder Archostemata [1]. The phylogenetic position of the family has been the subject of much controversy [2-8]. Based on differences of the aedeagus of *Omma stanleyi* Newman, 1839 [9] and *Tenomerga mucida* (Chevrolat, 1829), Ommatidae was first proposed by Sharp and Muir [8]. Crowson [2] placed the two recent ommatid genera in separate tribes, Ommatini and Tetraphalerini, and in 1976, he elevated both to family level [4,5]. In 1995, Lawrence and Newton [10] found the enclosure of sensilla in a deep sensorial cavity on the apical maxillary palpomere as an additional character uniting *Omma* and *Tetraphalerus*. This cavity is absent in Cupedidae. Based on this observation, Lawrence [7] discussed the relationships of the two genera to Cupedidae. He suggested to elevate Ommatinae to Ommatidae and described the features of this family. To date, a systematic position of Ommatidae within Archostemata is generally accepted [11-14].

Ommatidae is one of the “most ancestral” extant beetle families and a very small group. It includes the two extant genera, *Omma* Newman, 1839 [9] and *Tetraphalerus* Waterhouse, 1901 [15], with a total of six extant species. Their distribution is restricted to subtropical and more or less arid regions of the southern hemisphere. In contrast to the very limited range of the genera today, the recorded distribution in the Mesozoic was much broader. In addition to well preserved ommatid fossils in Mesozoic Lagerstätten of the eastern part of Russia and central Asia [3,16-18], interesting and important fossils of the group were discovered not only at fossil sites in northeastern China [19], but also at sites in Spain [20] and other localities [2,21]. Up to now, thirteen fossil genera with more than 100 species of ommatids have been described [2,3,16-26]. However, their systematic position within Ommatidae remained obscure.

Recently we collected several well-preserved fossils from the Jiulongshan Formation of Inner Mongolia and the ‘Jianshangou Bed’ in the lower part of the Yixian Formation of western Liaoning Province, China, respectively. Based on these material, four new species, *Pareuryomma ancistrodonta* sp. nov., *P. cardiobasis* sp. nov., *Omma delicata* sp. nov., and *Tetraphalerus decorosus* sp. nov., are described herein. *O. delicata* sp. nov. is the first fossil record of the genus *Omma* in China. These attractive fossils provide a very good opportunity to study morphological transformations within the family, such as changes of body size and modifications of the antennae, prothorax and elytra, and induced us to carry out the phylogenetic investigation of extant and extinct groups of this ancestral group of beetles. The morphological data obtained from the new specimens in combination with characters of other groups of Ommatidae and other archostematan taxa were used in a formal character evaluation. It is the first phylogenetic analysis of extant and extinct lineages of Ommatidae. Our study of these new ommatid species and the phylogenetic results provide a new understanding of the origin and evolution of Ommatidae. They also enable us to update the phylogenetic relationships of the genera of Ommatidae and to present a new classification for the group.

## Methods

All specimens were collected from a fossil locality near Daohugou Village, Shantou Township, Ningcheng County, Inner Mongolia, China and the ‘Jianshangou Bed’ in the lower part of the Yixian Formation of western Liaoning Province, China, respectively. The age of the Daohugou beds was confirmed as the Middle Jurassic by extensive evidence [27]. Recent studies have confirmed the age of the Yixian Formation as Early Cretaceous. The precise age is most likely restricted to 129.7–122.1 Ma (Barremian to Early Aptian) [28,29].

This study is based on several specimens housed in the Key Lab of Insect Evolution & Environmental Changes, the College of Life Sciences, Capital Normal University, Beijing, China (CNUB; Dong Ren, Curator).

The specimens were examined with a LEICA MZ12.5 dissecting microscope and illustrated with the aid of a drawing tube attached to it. The inking was performed with Adobe illustrator CS5 graphics software. Photographs were taken using a Nikon D300s digital camera connected to a ZEISS Discovery V12 dissecting microscope, and processed using Adobe Photoshop. The systematic arrangement used here mainly follows Lawrence [7]. The body length was measured from the anterior clypeal margin to the apex of the abdomen. The body width was measured at the base of the elytra. The length of the elytra was measured from the horizontal anterior margin of the base to the apex.

To comply with regulations of the International Code of Zoological Nomenclature (ICZN), we have deposited paper copies of the above article at the Natural History Museum, London; the American Museum of Natural History, New York; the Muséum National d’Histoire Naturelle, Paris; the Russian Academy of Sciences, Moscow; and the Academia Sinica, Taipei.

(a) *Taxon sampling and characters used in the phylogenetic analysis*

One species of Permocupedidae (*Permocupes sojanensis* Ponomarenko, 1963) [3,30] and one species of the cupedid subfamily Triadocupedinae (*Platycupes dolichocerus* Ponomarenko, 1966) were used as outgroup taxa. The outgroup selection was based on a family level phylogeny of Archostemata [11]. The ingroup consists of twenty-six terminal taxa including members of two extant genera, *Omma* and *Tetraphalerus*, and species of thirteen extinct genera. Due to the very incomplete preservation of the adults of *Blapsium egertoni* Westwood, 1854 [24,31], this taxon was not included in the analyses. Only adult characters are used as only one tentative larva of *Omma* is known [7] and no larvae of extinct ommatid species.

Taxon

Permocupedidae Martynov, 1933

Permocupes sojanensis Ponomarenko, 1966

Cupedidae Lacordaire, 1857

Platycupes dolichocerus Ponomarenko 1966

Ommatidae Lawrence, 1999

Omma delicata Tan, Ren, et Yang. MS

*Omma stanleyi Newman, 1839 (Recent)

*Omma mastersii Macleay, 1869 (Rencent)

*Omma gobiense* Ponomarenko, 1997

*Pareuryomma tylodes* Tan, Ren, *et* Shih, 2006

*Pareuryomma ancistrodonta* Tan, Ren *et* Yang*.* MS

Pareuryomma cardiobasis Tan, Ren et Yang. MS

Cionocoleus cervicalis Tan, Ren, et Shih, 2007

Odontomma trachylaena Ren, Tan et Ge, 2006

Brochocoleus applanatus Tan et Ren, 2009

Liassocupes parvus Zeuner 1962

Tetraphalerus decorosus Tan, Ren et Yang. MS

*Tetraphalerus bruchi Heller, 1913 (Rencent)

*Tetraphalerus wagneri Waterhouse, 1901 (Recent)

Tetraphalerus brevicapits Delclòs and Ponomarenko, 2000

Tetraphalerus glabratus Ponomarenko, 1997

Notocupoides triassicus Ponomarenko, 1966

Rhabdocupes longus Ponomarenko, 1966

Zygadenia viridis Sorinao and Delclòs, 2006

Notocupes pingi Ponomarenko, 2010

Notocupes nigrimonticola Ponomarenko, 1968

Eurydictyon conspicuum Ponomarenko, 1969

Amblomma psilata Tan, Liu et Ren, 2005

Amblomma porrecta Tan, Ren, et Shih, 2006

*Lithocupes punctatus* Ponomarenko 1969

*Tetraphalerites oligocenicus* Crowson, 1962

### List of characters and character states

The set of characters used in the analyses is mainly based on a list presented in Beutel *et al.*[12], a study focused on the systematic position of *Tetraphalerus* in Archostemata. More detailed information on character states was gained from the observation of specimens and original descriptions in the literature [2,3,7,8,17,18,20,23,24,30-35].

0. **The dorsal head surface longitudinal bulge or keel: (0) present; (1) absent.** A distinct and sharp longitudinal bulge or keel is present on the dorsal side of the head of *Notocupoides*, *Notocupes*, *Rhabdocupes*, *Zygadenia*, *Eurydictyon* and *Amblomma*[33], and also in *Permocupes* and *Platycupes*[3].

1. **Posteromesal dorsal protuberance of the head**[12]**: (0) present; (1) absent.** A moderately convex protuberance is present in the cupedid genus *Platycupes*, in *Tetraphalerus*[12] and *Notocupoides*, *Eurydictyon*, *Notocupes*, *Rhabdocupes*, *Zygadenia*, *Lithocupes*, *Liassocupes* and *Amblomma*[3,19]. It is not recognizable in extant species of *Omma* and the fossil genera *Cionocoleus*, *Pareuryomma*, *Odontomma* and *Brochocoleus*[19].

2. **Median epicranial (=coronal) suture: (0) present; (1) absent.** The epicranial suture is not visible and apparently absent in *Omma*[7,12] and *Cionocoleus*[36]. It is distinct in the genera *Notocupoides, Notocupes, Rhabdocupes, Zygadenia, Eurydictyon, Amblomma*, *Tetraphalerus*, *Pareuryomma*, *Brochocoleus*, *Permocupes* and Cupedidae [3,12,19,33].

3. **Head with ventrolateral antennal grooves: (0) absent; (1) present.** The presence of lateral longitudinal grooves on the ventral side of the head is an autapomorphy of *Tetraphalerus*[12]. They are absent in *Omma* and Cupedidae, and are also missing in fossil ommatid taxa.

4. **Gular sutures: (0) complete, reaching hind margin of head capsule; (1) incomplete, not reaching hind margin of head capsule; (2) absent.** The gular sutures of *Platycupes* and *Permocupes*, and the fossil ommatid genera *Eurydictyon*, *Notocupoides*, *Notocupes*, *Rhabdocupes*, *Zygadenia* and *Amblomma* reach the hind margin of the head capsule [3,33]. They are incomplete in *Omma*[6,7]. The gular sutures are not recognizable in extant species of *Tetraphalerus*[12].

5. **Shape of gula: (0) sutures not converging posteriorly; (1) sutures converging posteriorly.** Diverging in *Platycupes* and some fossil ommatids such as *Notocupoides*, *Rhabdocupes*, *Notocupes*, *Zygadenia* and *Amblomma*, and also in *Permocupes*[3,33]. Converging in *Omma*[6].

6. **Frontoclypeal suture: (0) present; (1) absent.** Usually absent in extant Archostemata [12], but present in *Notocupoides*, *Rhabdocupes*, *Notocupes*, *Eurydictyon* and *Amblomma*, and also in *Permocupes*[3,33].

7. **Ratio of labrum to clypeus: (0) labrum strongly transverse, as wide as clypeus (ratio 1); (1) labrum distinctly narrower than clypeus (ratio less than 1).** The labrum of *Amblomma*, *Brochocoleus*, *Pareuryomma*, *Odontomma*, *Cionocoleus* and *Lithocupes*[3,19] is distinctly narrower than the clypeus [3], whereas the labrum of *Tetraphalerus* and *Permocupes* is strongly transverse and as wide as the clypeus [12]. This character is not visible in some fossil taxa. The labrum fused with the clypeus in the genus *Omma*.

8. **Anterior margin of labrum: (0) distinctly convex; (1) truncate.** The anterior margin of the labrum is distinctly convex in the genera *Notocupoides*, *Rhabdocupes*, *Notocupes*, *Eurydictyon*, *Amblomma*, *Lithocupes*, *Cionocoleus*, *Odontomma*, and *Omma*[3,7,33], and also in *Permocupes* and *Platycupes*[3]. It is truncate in the genus *Pareuryomma*, *Brochocoleus* and *Tetraphalerus*[12].

9. **Length of antenna: (0) reaching mesothorax posteriorly; (1) not reaching hind margin of prothorax posteriorly.** The antennae are strongly elongated in Cupedidae, but moderately long or even short in most members of Ommatidae [7].

10. ** Length of pedicel: (0) not distinctly shortened; (1) distinctly shortened.** Not distinctly shortened, nearly as long as the third antennomere in *Notocupoides*, *Eurydictyon*, *Pareuryomma* and also in *Permocupes*[3,12,19]. It is greatly shortened in the other taxa under consideration.

11. ** Length of third antennomere: (0) not elongated; (1) distinctly elongated.** The third antennomere of *Permocupes* and *Platycupes*, and of the genera *Notocupoides*, *Rhabdocupes*, *Notocupes*, *Zygadenia*, *Eurydictyon* and *Pareuryomma* is moderately long, about as long as the fourth. It is distinctly elongated, longer than the fourth antennomere in the other ommatid tribes [12].

12. ** Apical flagellomere: (0) apically rounded, appearing inflated; (1) parallel-sided.** The apical flagellomere of *Pareuryomma* (Figure 1B), *Tetraphalerus decorosus* (Figure 2B) and Permocupedidae [3] is apically rounded and appears inflated. It is parallel-sided and slender in the other genera of Ommatidae and Cupedidae.

**Figure 1:**
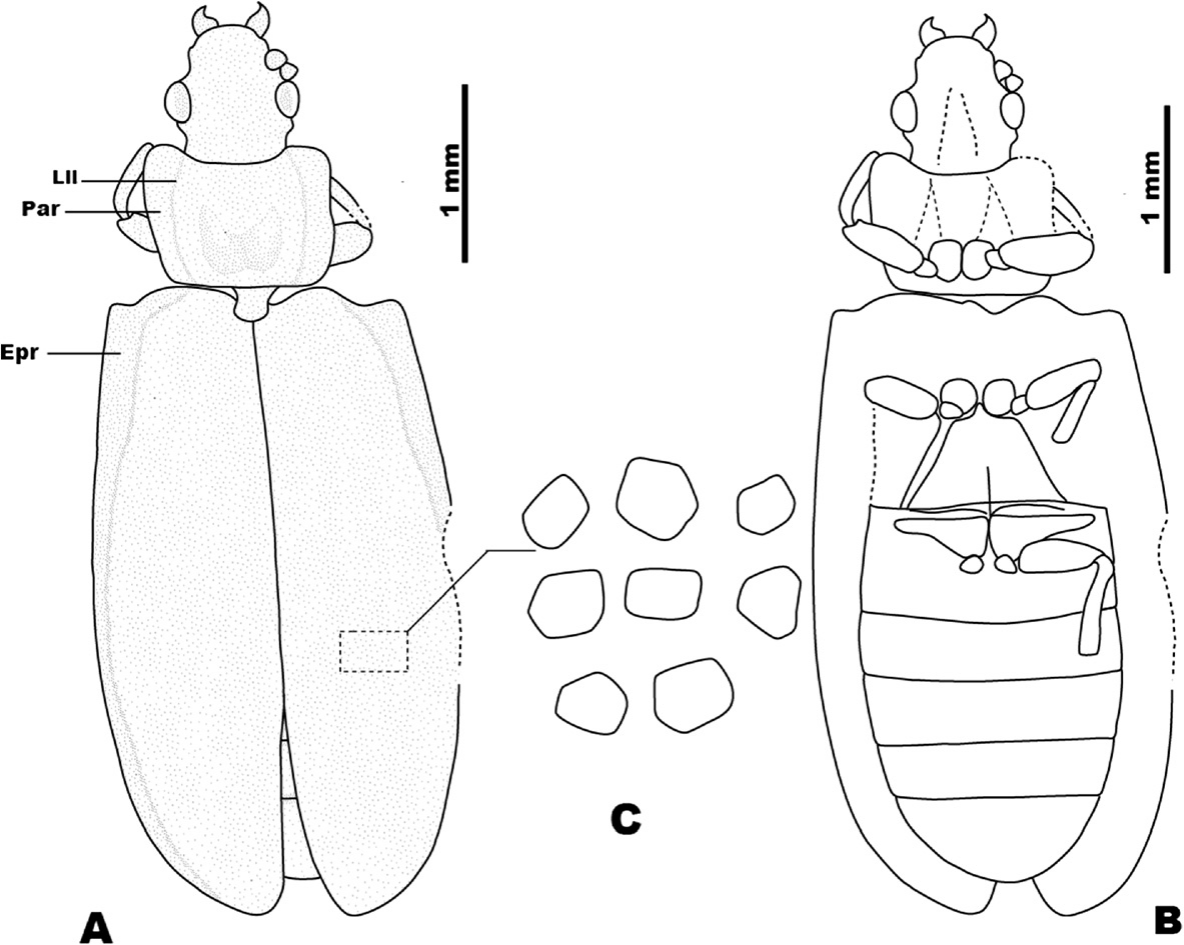
***Pareuryomma cardiobasis *sp. nov., holotype, No. CNU-C-NN2010809.A**, line drawing, dorsal view. **B**, line drawing, ventral view. Abbreviations: Aea = Anterior elytral angle; Epr = Epipleural rim; Lll = Lateral longitudinal line; Par = Paranotum.

**Figure 2:**
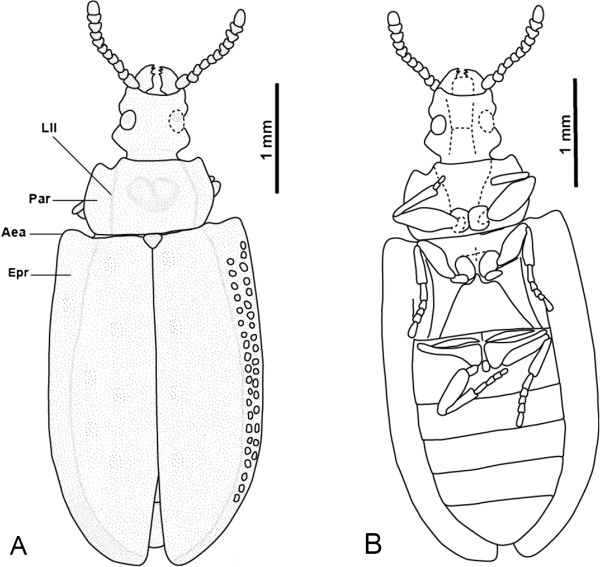
***Tetraphalerus decorosus *sp. nov., holotype, No. CNU-C-NN2010812.A**, line drawing, dorsal view. **B**, line drawing, ventral view. **C**, line drawing of elytral cells. Abbreviations: Epr = Epipleural rim; Lll = Lateral longitudinal line; Par = Paranotum.

**Figure 3:**
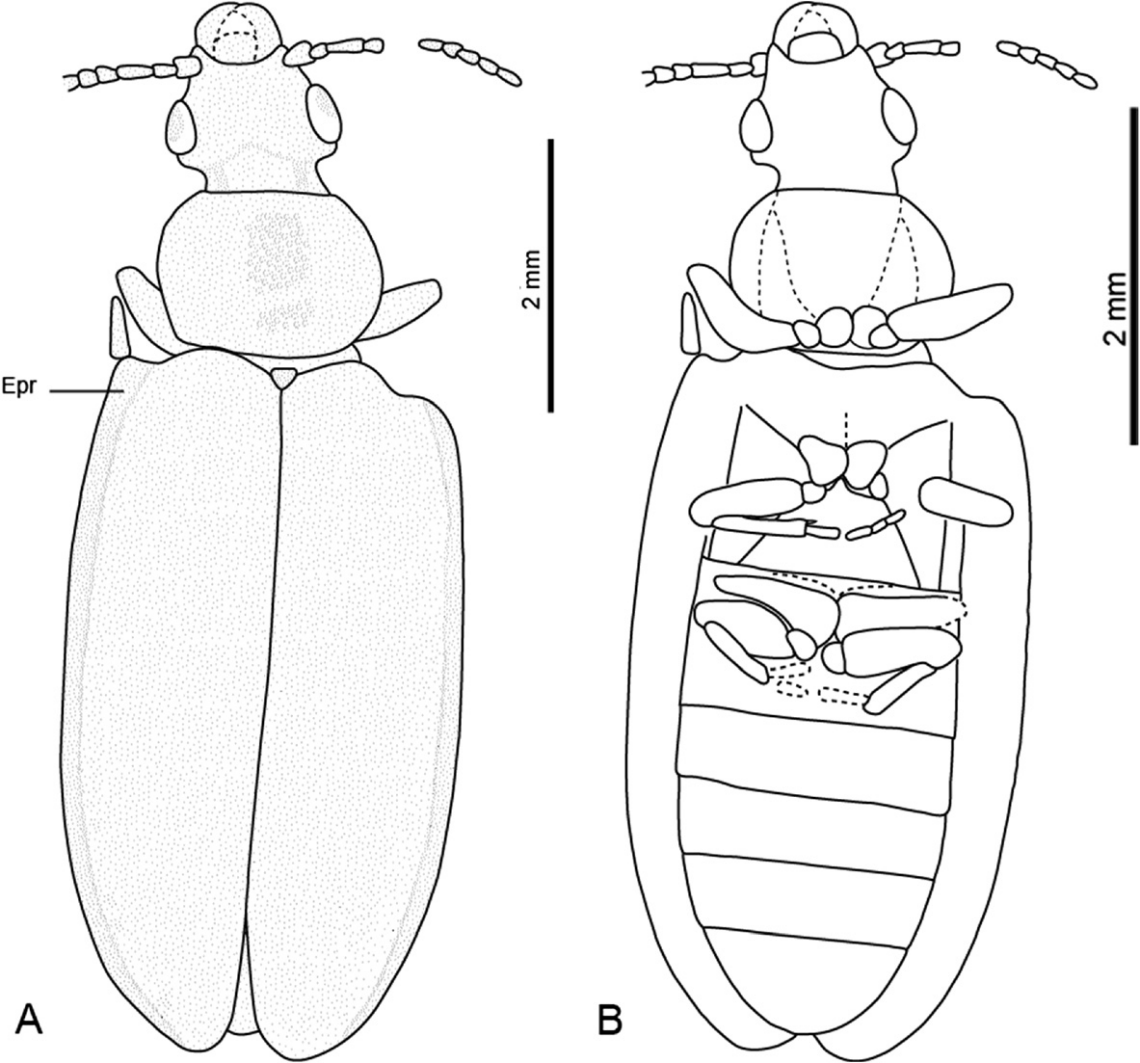
***Pareuryomma ancistrodonta *sp. nov. holotype, No. CNU-C-NN2010808.A**, line drawing, dorsal view. **B**, line drawing of ventral view. **C**, line drawing, elytral cells. Abbreviations: Epr = Epipleural rim; Lll = Lateral longitudinal line; Par = Paranotum.

**Figure 4:**
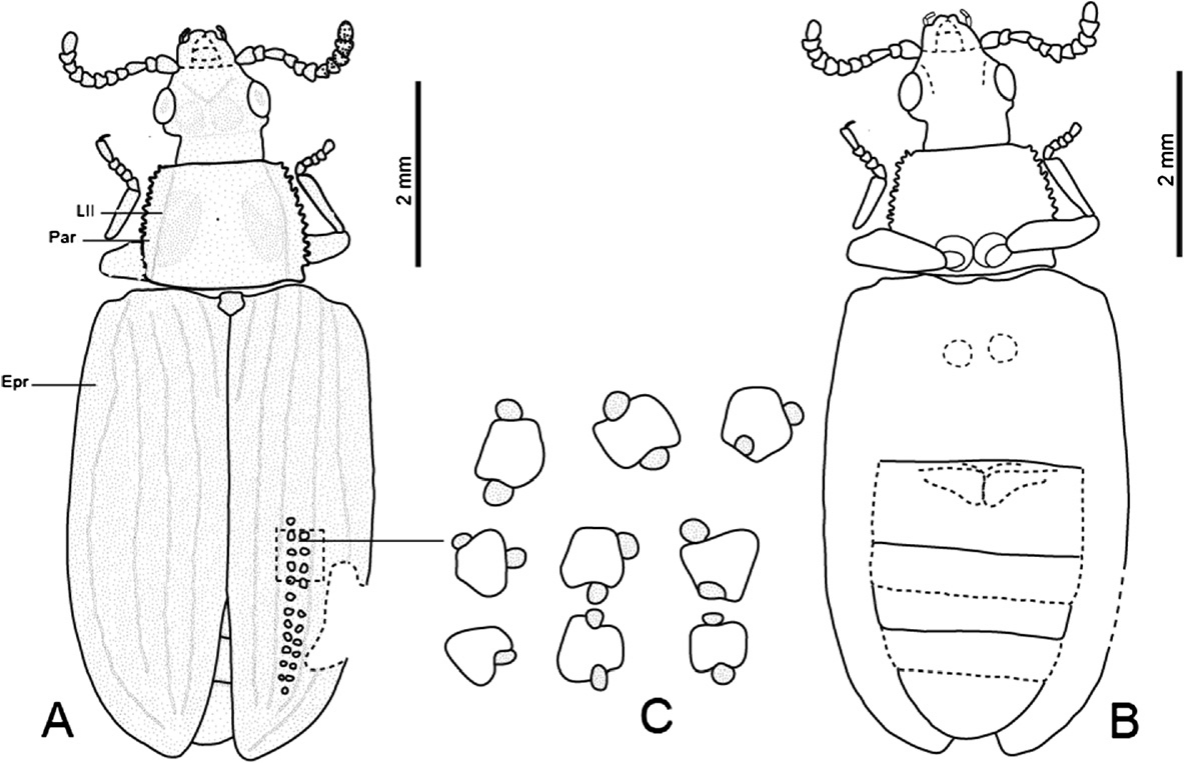
***Omma delicata *sp. nov., holotype, No. CNU-C-NN2010813.A**, line drawing, dorsal view. **B**, line drawing, ventral view. Abbreviation: Epr = Epipleural rim.

13. ** Length of mandible: (0) short or moderately long, largely covered by labrum in repose; (1) very elongate and protruding in resting position.** Moderately long or short and largely covered by the labrum in the resting position in most extant Archostemata and most of the fossil taxa. Exceptionally long and distinctly protruding in *Tetraphalerus*[12], *Cionocoleus*[19], *Omma gobiense* Ponomarenko, 1997

14. ** Cutting edge of mandible: (0) teeth horizontally arranged in longitudinal direction or only one apical tooth; (1) with vertically arranged teeth.** The presence of three vertically arranged mandibular teeth is a characteristic feature of extant Ommatidae [7,12] and Micromalthidae [12]. The cutting edge is horizontal in Cupedidae as in most fossil ommatid beetles.

15. ** Pronotum: (0) narrowing anteriorly; (1) narrowing posteriorly.** The pronotum is narrowing posteriorly in *Pareuryomma* (Figures 1A and 3A), but anteriorly in the other genera.

16. ** Surface convexities of the pronotum: (0) present; (1) absent.** Only absent in *Omma*, *Cionocoleus*, and *Odontomma*[19]. Present in Cupedidae and other ommatids [3,6].

17. ** Lateral pronotal projection (ventrally corresponding with pronotal epipleura): (0) as wide as or wider than one-fourth of the width of the main part of the pronotum; (1) narrower than one-fourth of the width of the main part of the pronotum.** The paranota of *Notocupoides*, *Rhabdocupes*, *Notocupes*, *Zygadenia*, *Eurydictyon*, *Amblomma*, *Cionocoleus*, *Pareuryomma*, *Brochocoleus*, *Odontomma*, *Lithocupes* and *Platycupes* are widened [3,19,36].

18. ** Procoxae: (0) separated; (1) adjacent.** Adjacent in Ommatidae (Figure 4B). Distinctly divided in Cupedidae and *Permocupes*[3].

19. ** Length ratio of the last to the penultimate abdominal ventrites: (0) less than 1.5 times; (1) more than 1.5 times.** The last abdominal ventrite is longer than the penultimate one in *Liassocupes*, *Notocupoides*, *Rhabdocupes*, *Zygadenia*, *Eurydictyon*, *Lithocupes* and *Amblomma*[3,19], and also in the cupedid genus *Platycupes*. It is even as long as the penultimate and antepenultimate ventrite in *Tetraphalerus wagneri*. (Hörnschemeyer T: *Die phylogenic der Archostemata (Insecta: Coleoptera) und die evolution der flügelbasis der holometabolen insekten,*unpublished.

20. **Arrangement of abdominal sterna: (0) abutting, not overlapping; (1) posterior margin overlapping with anterior part of following ventrite.** The posterior margin of the abdominal ventrites is overlapping with the anterior ones in Cupedidae and the genera *Notocupoides*, *Rhabdocupes*, *Notocupes*, *Zygadenia*, *Eurydictyon* and *Amblomma*[3], whereas the ventrites are abutting in *Brochocoleus*, *Odontomma*, *Pareuryomma*, *Omma*, *Cionocoleus* and *Tetraphalerus*.

21. ** Length of mesofemur: (0) extending well beyond side margins of body; (1) not extending beyond side margins of body.** The mesofemur of *Omma*, *Cionocoleus*, *Brochocoleus*, *Odontomma*, *Notocupoides*, *Rhabdocupes*, *Notocupes*, *Zygadenia*, *Eurydictyon* and *Lithocupes* is extending well beyond the lateral margin of the body, whereas it is short in the genera *Tetraphalerus*[2], *Amblomma* and *Pareuryomma*.

22. **Main elytral veins: (0) all longitudinal veins end on sutural margin before elytral apex; (1) only 2 sutural main veins meet before elytral apex.** Two longitudinal main veins meeting before the elytral apex are present in *Rhabdocupes*, *Notocupes*, *Zygadenia*, *Eurydictyon*, *Amblomma* and *Brochocoleus*[3,19].

23. ** Epipleural rim near basal part: (0) narrow, one row of cells; (1) more than one row of cells.** More than one row of cells is present in the basal part of the epipleural rim of *Pareuryomma*, *Odontomma* and *Brochocoleus*[19].

24. ** Elytra with cells (window punctures) on disk: (0) present; (1) absent.** The cells are absent or very indistinct on the disk in *Cionocoleus* and *Tetraphalerites*, and the extinct species of *Omma* and *Tetraphalerus*[6,24,36].

25. ** Size of window punctures: (0) enlarged, separated by very distinct rib-like ridges; (1) small.** The window punctures of *Eurydictyon*, *Notocupes*, *Notocupoides*, *Amblomma* and Triadocupedinae are about 4 times as large as in the other taxa under consideration [3] and separated by conspicuous rib-like ridges [3].

26. ** Regular row of large window punctures along external elytral margin: (0) present; (1) absent.** A conspicuous regular row of large window punctures is present along the external elytral margin in *Eurydictyon*, *Notocupes* and *Notocupoides*, *Amblomma*, *Lithocupes*, *Liassocupes*, *Pareuryomma*, *Odontomma* and *Brochocoleus,* and also in Triadocupedinae [3].

27. ** Pentagonal enlarged window punctures along elytral suture: (0) absent; (1) present.** Pentagonal enlarged window punctures are present along the elytral suture in *Eurydictyon* and *Notocupoides*[3].

(b)Parsimony analysis

The data matrix (see Additional file 1) used for the analysis contains 28 taxa (Table 1) and 28 morphological characters. All characters were equally weighted and non-additive. The matrix was modified in WinClada [37] and analyzed with NONA version 1.5 (options set to hold 10000 trees, perform 1000 replications with one starting tree replication, and the multiple TBR + TBR search strategy) [37], and TNT (algorithm: traditional search; starting trees: Wagner trees, 100 replicates; swapping algorithm: tree bisection reconnection) [38], respectively. Nodal support for the cladogram was assessed using Bremer support (BS) [39,40] values in TNT. Kirejtshuk *et al.*[41] proposed some synonyms within Ommatidae. Herein we conducted the topological constraints to determine the obscure interrelationships among *Tetraphalerus* + *Odontomma* and *Pareuryomma* + *Notocupes* using PAUP v4b10. Three topological constraints were imposed in cladistic analysis enforced the monophyly of *Tetraphalerus* + *Odontomma* and *Pareuryomma* + *Notocupes* respectively according to Kirejtshuk’s hypothesis.

**Table 1:** **Geological distribution of the known fossil species of *Omma***

**Age**	**Species**	**Location and horizon**	**Reference**
K_1_	*O. sibiricum* Ponomarenko, 1966	Baissa, West Transbaikalia Russia; Zazinskaya **Fm.**	Ponomarenko, 1966
	*O. antennatum* Ponomarenko, 1997	Bon-Tsagan, Mongolia; Khurilt sequence	Ponomarenko, 1997
	*O. gobiense* Ponomarenko, 1997	Khoutiin-Khotgor, Mongolia; Ulan-Ereg **Fm.**	Ponomarenko, 1997
	*O. brevipes*, Deichmüller, 1886	Solnhofener, German; Solnhofener **Fm.**	Deichmüller, 1886
	*O. zitteli* Oppenheim, 1888	Solnhofener, German; Solnhofener **Fm.**	Oppenheim, 1888
J_3_-K_1_	*O. pilpsim* Ponomarenko, 1964	Karatau, Kazakhstan; Karabastau **Fm**.	Ponomarenko, 1964
	*O. aberratum* Ponomarenko, 1968	Karatau, Kazakhstan; Karabastau **Fm.**	Ponomarenko, 1968
	*O. jurassicum* Ponomarenko, 1968	Karatau, Kazakhstan; Karabastau **Fm.**	Ponomarenko, 1968
J_2_-J_3_	*O. altajense* Ponomarenko, 1997	Bakhar, Mongolia; Togo-Khuduk sequence	Ponomarenko, 1997
	*O. delicata* sp. nov.	Daohugou, Inner Mongoliia Jiulongshan **Fm.**	this study
J_1_	*O. avus* Ponomarenko, 1969	Issyk-Kul’, Kyrgyzstan; Dgil’skaya **Fm.**	Ponomarenko, 1969
J_1_-T_3_	*O. liassicum* Crowson, 1962	Brown’s wood, Warwickshire, England; Rhaetian Age**.**	Crowson, 1962

The systematic position of the taxa is based on original descriptions. T_3_ —Late Triassic; J_1_—Early Jurassic; J_2_—Middle Jurassic; J_3_—Late Jurassic; K_1_—Early Cretaceous; E_3_-Oligocene; Fm.—Formation.

## Results

### Description of the specimens

Order **Coleoptera Linnaeus, 1758**

Family Ommatidae Sharp *et* Muir, 1912

Subfamily Ommatinae Sharp *et* Muir, 1912

Tribe **Ommatini Sharp*et*Muir, 1912**

Genus ***Pareuryomma*Tan, Ren, Shih*et*Ge, nom. nov.**

***Pareuryomma*Tan, Ren, Shih*et*Ge, 2006 (nect. Stein, 1899) nom. nov.**

***Pareuryomma*nom. nov., for*Euryomma*Tan, Ren, Shih*et*Ge 2006 (Coleoptera: Archostemata) nect. Stein, 1899 (Diptera: Fanniidae).**

#### Type species

*Pareuryomma tylodes* (Tan, Ren, Shih *et* Ge, 2006) **comb. nov.**

#### Included species

*Pareuryomma tylodes* (Tan, Ren, Shih *et* Ge, 2006) **comb. nov.**, *P. ancistrodonta* sp. nov., and *P. cardiobasis* sp. nov.

#### Emended diagnosis

Whole body surface with large round tubercles, especially on head and prothorax; dorsal head surface without macroscopic tubercles. Mandibles prominent, incurved, with vertically arranged teeth. Antennae not reaching posterior margin of prothorax, 11-segmented, antennomeres 7–10 very slightly extended, resulting in a slightly serrated appearance of this part of antenna; apical flagellomere short and wide, apically rounded, appearing inflated. Pronotum widest anteriorly, narrowing posteriorly; anterior angles moderately produced. Elytra very distinctly convex basally, flattened towards apex, with 8 rows of cells on disc; epipleural space wide anteriorly, narrowing towards apex; with 2 rows of cells in the proximal half and one row in the apical half; principal longitudinal veins parallel to the sutural margin. Abdominal sternites arranged in one plane. First visible ventrite longest, last visible ventrite (=sternite VII) about 1.4 times as long as preceding one.

#### Note

The emended diagnosis is based on the type species and the new materials.

#### Occurrence

Type species and *Pareuryomma cardiobasis* sp. nov. are from the Early Cretaceous Yixian Formation, near Chaomidian Village, Beipiao City, Liaoning Province, China. *Pareuryomma ancistrodonta* sp. nov. is from the Middle Jurassic Jiulongshan Formation, Daohugou, Ningcheng, Inner Mongolia, China.

#### Remarks

The genus *Euryomma* was erected by Tan, Ren, Shih *et* Ge [34]. However, the name has already been used previously to describe flies of the family Fanniidae (*Euryomma* Stein, 1899 (type species: *Anthomyia peregrine* Meigen, 1826 (= *Euryomma hispaniense* Stein, 1899)) [42]. Consequently, the beetle genus *Euryomma* became the homonymy of *Euryomma* of the family Fanniidae. Based on the “International Code of Zoological Nomenclature (fourth edition)”, we amend the genus name from “*Euryomma*” to “*Pareuryomma*”.

Kirejtshuk *et al.*[41] considered *Pareuryomma* as a synonym of *Notocupes* Ponomarenko, 1964. The new fossil specimens clearly show that this genus is distinctly different from *Notocupes*. *Pareuryomma* can be distinguished from *Notocupes* by the following characters: dorsal head surface without longitudinal bulge or keel; antennae moniliform, antennomeres 7–11 widened distally; pronotum widest anteriorly, narrowing posteriorly; abdominal ventrites arranged in one plane, not overlapping.

### *Pareuryomma ancistrodonta* sp. nov. 

#### Diagnosis

Mandible with one apical tooth; temples shorter than eyes; anterior edge of pronotum distinctly curved inwards; anterolateral edge of pronotum rounded; central disc with 2 flat elevations.

#### Etymology

Name derived from the Greek compound word ‘*ancistrodonta*’, *-a*, *-um*, (with falcate tooth), referring to the sickle-shaped mandible.

#### Type material

Holotype: a well-preserved adult with body, elytra, and parts of legs and antennae. Registration No. CNU-C-NN2010808, housed in Key Lab of Insect Evolution & Environmental Change, Capital Normal University, Beijing, China.

#### Type locality and horizon

Daohugou Village, Shantou Township, Ningcheng County, Inner Mongolia, China; Jiulongshan Formation, Middle Jurassic (Bathonian- Callovian boundary).

#### Description

**Figure 5:**
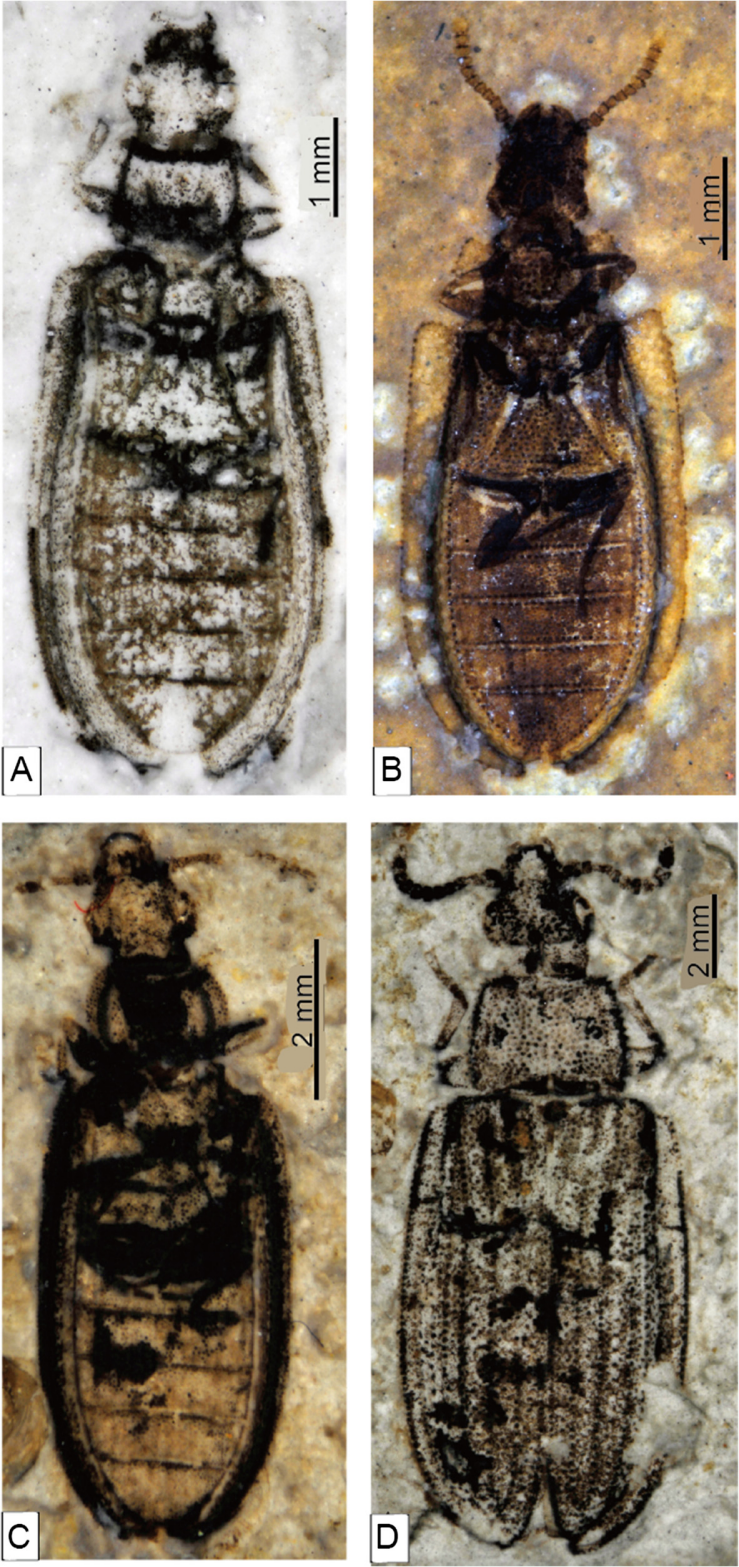
**Photograph of fossil ommatids.A**, *Pareuryomma ancistrodonta* sp. nov. holotype, No. CNU-C-NN2010808. **B**, *Pareuryomma cardiobasis* sp. nov. holotype, No. CNU-C-NN2010809. **C**, *Omma delicata* sp. nov., holotype, No. CNU-C-NN2010813. D, *Tetraphalerus decorosus* sp. nov., holotype, No. CNU-C-NN2010812.

Body small, surface densely covered with tubercles (Figure 5A).

*Head capsule:* with distinctly protruding compound eyes approximately as wide as long; distinctly narrower anteriorly and posteriorly than at ocular region and temples; distinctly prognathous; without raised tubercles or longitudinal bulge or keel on dorsal surface; anterior margin very slightly convex; frontoclypeal suture not recognizable; temples short, about 0.3 times as long as eyes, moderately projecting laterally (Figure 3A). Compound eyes at middle region of head approximately round in outline, distinctly convex and protruding laterally; neck region moderately constricted, slightly shorter than compound eyes. Gula sub-rectangular, narrowing anteriorly; gular suture reaching posterior margin of head, widely separating genal regions on ventral side (Figure 3B).

*Head appendages:* labrum not recognizable. Antennae inserted laterally, at about midlength between anterior margin of head capsule and anterior margin of eyes; scape slightly wider than long; pedicel shorter than scape; flagellum not preserved. Mandibles short, with fairly broad proximal part and with one apical tooth (Figure 3A). Ventral mouthparts not recognizable.

*Pronotum:* subparallel, slightly narrowing posteriorly; distinctly wider than head; about 2/3 as long as wide at anterior edge; about 3/5 times as long as width at posterior edge; anterior pronotal edge moderately concave; posterior edge straight; anterolateral edge rounded, moderately produced; posterior angles rounded; lateral margins almost straight, very slightly curved; lateral longitudinal line separating lateral projection from slightly convex main part of pronotum distinct; lateral projection (paranotum) slightly widened; central disc with two flat elevations (Figure 3A).

*Scutellar shield:* exposed, shield-like, distinctly longer than wide; lateral margins concave, slightly widening anteriorly.

*Elytra:* both elytra together about 2 times as wide as maximum width of prothorax; maximum width at elytral midlength; individual elytron 3.2 times as long as wide; very strongly pronounced dorsolateral ridges and sharply pronounced lateral edge enclose flat or even slightly concave epipleural rim; elytral disc enclosed by dorsolateral ridges, also flat or slightly concave except for anteriormost part; epipleural rim widening in anterior half, lacking distinct cells, narrowing towards apex; dorsolateral ridges anteriorly connected with elytral shoulders; main veins of elytra similar to intermediate ones (Figure 3A); elytral cells (=window punctures) moderately distinct, small; regular rows of large cells along lateral elytral margin absent (Figure 3C); elytra reaching slightly beyond abdominal apex; appearing dehiscent in posterior third (possibly an artifact of compression during fossilization).

*Thoracic venter:* prothoracic notopleural suture straight and diverging posteriorly; anapleural cleft converging posteriorly towards procoxae; both sutures enclose triangular, anteriorly narrowing pleura, which meet the anterior prothoracic margin at a narrow point; prosternal process absent; protrochantin not recognizable. Individual structures of mesoventrite not recognizable. Metaventrite trapezoidal, distinctly narrowing anteriorly; posterior margin 1.8 times as wide as length at midline; anterior margin distinctly narrower than posterior margin, about as wide as mesocoxae combined; mesocoxae separated by short, rounded median process of metaventrite; metathoracic discrimen and metakatepisternal suture present; metatrochantin present and exposed between hind margin of katepisternum and coxa. Individual structures of metapleuron not recognizable; metanepisternum apparently broad anteriorly and narrowing towards end of segment (Figure 3B).

*Legs:* procoxae medially adjacent, medium-sized, cone-shaped but rounded apically; protrochanter small, slightly oblong; profemora short, widening distally, with strongly convex posterior margin, distinctly thicker than proximally curved and slender protibiae and slightly longer; mesocoxae slightly smaller than procoxae, nearly circular; mesotrochanter nearly oblong; mesofemora short, not reaching lateral margin of elytra; parallel-sided, thicker than mesotibia and about equally long; metacoxae transverse, distinctly reaching beyond lateral margin of metaventrite; triangular, without metacoxal plates; posteromesal apices rounded, far from posterior margin of first visible abdominal ventrite; metatrochanter small, nearly triangular; metafemur short, not extending beyond lateral margins of body, proximally distinctly thicker than distally; metatibia longer than metafemur; curved proximally, subparallel, widening very slightly distally.

*Abdomen:* with five visible ventrites; narrowing towards apex from the base of ventrite 3; first ventrite longer than other ones; last ventrite (=sternite VII) 1.5 times as long as the previous one; hind margin evenly rounded (Figure 3B).

Dimensions (mm): body length 7.5, width 2.8, head length 1.1, width 1.2, anterior edge of pronotum 2.0, posterior edge of pronotum 1.5, pronotal width 1.1, elytral length 5.3, width 1.4.

#### Comments

*Pareuryomma ancistrodonta* sp. nov. clearly belongs to the genus *Pareuryomma* indicated by the following characteristics: pronotum widest anteriorly, narrowing posteriorly; epipleural space of elytron wide with 2 rows of cells in the proximal half and narrowing towards the apex. The new species differs from the type species *P. tylodes* in several features: temples shorter than the eyes, anterior pronotal angle rounded. It can distinguish from *P. cardiobasis* sp. nov. in the specific shape of the mandibles with a single apical tooth.

### ***Pareuryomma cardiobasis*** sp. nov. 

#### Diagnosis

Mandibles stout, with a distinctly dilated and tridentate apical part; temples conspicuously projecting laterally, about as long as eyes; anterior margin of pronotum as long as posterior margin; pronotal hypomeron wide; central disc with cordiform elevation.

#### Etymology

Name derived from the Greek word ‘*cardiobasis*’, *-is*, *-e*, (heart-shaped), referring to the central disc of the pronotum with a large cordiform elevation.

#### Type material

Holotype: a well-preserved adult with body, elytra, legs and antennae. Registration No. CNU-C-LB2010809, housed in Key Lab of Insect Evolution & Environmental Change, Capital Normal University, Beijing, China.

#### Type locality and horizon

Near Chaomidian Village, Beipiao City, Liaoning Province, China, Yixian Formation (Early Cretaceous).

#### Description

Body small, flattened, densely covered with large tubercles (Figure 5B).

*Head capsule:* slightly shorter than maximum width at temples; upper surface more or less flattened, without raised tubercles or other prominent structures; anterior margin distinctly concave; frontoclypeal suture not recognizable (Figure 1A). Compound eyes at middle region of head largely integrated in the contour of the head, scarcely protruding laterally; neck region distinct narrow. Gula sub-rectangular; gular ridges reaching posterior margin of head, widely separating genal regions on ventral side (Figure 1B).

*Head appendages:* labrum not recognizable. Antennae inserted laterally; clavate, not reaching posterior margin of prothorax, distally widening; scape as long as last antennomere; pedicel wider than long, nearly as wide as scape; third antennomere not longer than second and fourth combined; antennomeres 7–10 short, distinctly broader than antennomeres 3–6; apical antennomere short and wide, appearing inflated. Labium with large submentum, slightly widening anteriorly, separated from gula by transverse suture; mentum not recognizable, apparently reduced; prementum large, plate-like; remaining elements of ventral mouthparts not visible (Figure 1A).

*Pronotum:* transverse, distinctly wider than head; widest at middle, slightly narrowing posteriorly; width at anterior margin about 1.5 times of length at midline; anterior pronotal edge along neck region almost straight, at most very slightly curved inwards, distinctly emarginated laterally, with rounded, moderately prominent anteriolateral edge; posterior angles completely rounded, not prominent; lateral longitudinal line separating lateral projection from slightly convex main part of pronotum moderately distinct; lateral pronotal projection (paranotum) distinctly widened; central disc bearing cordiform elevation (appearing as convexity in specimens with exposed ventral side) (Figure 1A).

*Scutellar shield:* exposed, triangular.

*Elytra:* disc flattened, only very slightly convex; both elytra together about 1.7 times as wide as maximum width of prothorax; individual elytron about 3 times as long as wide; maximum width at about midlength of elytra; pronounced dorsolateral ridges of elytra and sharply pronounced lateral edge enclosing flat or even slightly concave epipleural rim; elytral disc enclosed by dorsolateral ridges, also flat or slightly concave except for anterior-most part; dorsolateral ridges anteriorly connected with elytral shoulders; epipleural rim with two rows of cells in the proximal two thirds and one row in the distal third; main veins of elytra similar to intermediate ones; elytral cells (=window punctures) on disc small, indistinct; regular row of large cells along lateral margin absent; elytra reaching slightly beyond abdominal apex; appearing dehiscent in posterior third (possibly an artifact of fossilization) (Figure 1A).

*Thoracic venter:* prothoracic anapleural cleft converging posteriorly towards anterior procoxal margin; notopleural suture not visible; prosternal process absent. Mesoventrite with distinct discrimen; mesokatepisternal suture present as a distinct arched row of punctures. Metaventrite trapezoid, distinctly narrowing anteriorly; posterior margin 1.9 times as wide as length at midline; anterior margin slightly narrower than mesocoxae combined; mesocoxae separated by short, triangular, apically pointed process of metaventrite; metathoracic discrimen present but very short; metakatepisternal suture present; metatrochantins present and exposed between hind margin of katepisternum and coxa. Metanepisternum triangular, extensive, strongly widening anteriorly, and narrowing towards end of segment (Figure 1B).

*Legs:* procoxae nearly spherical, protruding, medially adjacent; protrochanter small, approximately triangular; profemora with convex anterior and posterior margins, distinctly widening in middle region, distinctly thicker than protibiae and slightly longer; protibia slender, slightly widening distally; two slender protarsomeres preserved; mesocoxae more conical, not protruding, posteriorly oriented, distinctly separated by mesokatepisternal region; mesotrochanter small, transverse; mesofemora with convex anterior and posterior margins, widening in middle region, distinctly thicker than mesotibia and slightly longer, not reaching beyond lateral margin of body; mesotibia subparallel, hind margin slightly convex; mesotarsus with 5 tarsomeres; tarsomeres 1 and 5 elongate, nearly equal in length; tarsomeres 2–4 distinctly shorter, only slightly longer than wide, nearly equal in length; metacoxae transverse, distinctly reaching beyond lateral margin of metaventrite; triangular, without metacoxal plates, posteromesal apices rounded, far from posterior margin of first visible abdominal ventrite; metatrochanter small, oblong; metafemur with very slightly convex anterior and posterior margin, short, not extending beyond lateral margin of body; metatibia very slightly widening distally, almost parallel-sided, shorter than metafemur; metatarsus 5-segmented; tarsomeres very similar to those of middle leg.

*Abdomen:* narrowing towards the apex from the base of ventrite 4; first abdominal ventrite longer than other ones, likely representing fused sternites II and III; last ventrite (=sternite VII) 1.35 times as long as previous one; hind margin evenly rounded.

Dimensions (mm): body length 6.6, width 2.5, head length 1.1, width 1.0, anterior edge of pronotum 1.5, posterior edge of pronotum 1.4, pronotal width 1.0, elytral length 4.5, width 1.4.

#### Comments

*Pareuryomma cardiobasis* sp. nov. differs from the type species in the following features: anterior margin of pronotum as long as the posterior margin, central pronotal disc bearing a cordiform elevation; from *P. ancistrodonta* in the presence of tridentate with perpendicular cutting edge of the mandible.

Genus ***Omma*Newman, 1839**

*Omma* Newman, 1839, Ann. Mag. Nat. Hist. 3:303; Peyerimhoff, 1902, Bull. Soc. Ent. Fr., p.330; Sharp and Muir, 1912, Trans. Ent. Soc. Lond., p. 521, 615, 632; Neboiss, 1959, Proc. Roy. Soc. Vic. 72:17; Crowson, 1962, Ann. Mag. Nat. Hist. 13(5): 147–157; Atkins, 1963, Can. Ent. 95: 140–162; Ponomarenko, 1964, Paleontol. Jour. 4: 45–55; Ponomarenko, 1966, Entomolog. Oboer. 1: 138–143; Ponomarenko, 1968 Nauka, Moscow. p. 118–138; Ponomarenko, 1969, Tru. Paleontol. Instit. A. N. SSSR, 125: 70–115; Ponomarenko, 1997, Paleontol. Jour. 31(4): 389–399; Lawrence, 1999, Inver. Taxon. 13:369–390; Ponomarenko, 2006, Paleontol. Jour. 40(1):90–99.

#### Type species

*Omma stanleyi* Newman, 1839; Recent.

#### Included species

Sixteen species have been described up to date. *O. stanleyi* Newman, 1839, *O. mastersii* Macleay, 1871, *O. sagitta* Neboiss, 1960 and *O. rutherfordi* Lawrence, 1999 are four extant species from Australia. The others are all fossil species from the Mesozoic of Siberia, Central Asia, Western Europe and China (shown in Table 1). In addition, *O. delicata* sp. nov. is described below.

#### Emended diagnosis

Body elongate, moderately flattened or almost cylindrical. Surface often tuberculate or rarely spinose. Head subquadrate to slightly elongate, always with distinct neck region; temples usually shorter than eye; antennal grooves absent. Antennae inserted laterally. Antennae filiform or somewhat moniliform, as long as or slightly shorter than head and prothorax together; antennomere 3 longest, as long as or longer than pedicel and antennomere 4 combined. Mandibles prominent, incurved. Labrum, clypeus, and frons fused. Pronotum subquadrate or slightly transverse, without lateral pronotal carinae; procoxal cavities contiguous. Legs moderately long and slender; tibiae usually not much longer than femora; mesofemora extending beyond side margins of body. Abdominal sternites arranged in one plane.

#### Note

The emended diagnosis is based on the type species and the new material.

***Omma delicata*** sp. nov. (Figure 1)

#### Diagnosis

Body small; head distinctly wider posteriorly behind compound eyes. Scape subtriangular, 1.2 times longer than pedicel, thicker than other antennomeres. Pronotum square, anterior and posterior angles of pronotum completely rounded, not prominent. Elytra with indistinct veins and cells; elytra 1.8 times as wide as prothorax.

#### Etymology

Name derived from the Latin word ‘*delicatus*’, *-a*, *-um* (delicate), referring to the relatively small size of the new species.

#### Type Material

Holotype: nearly complete adult ommatid with body, elytra, part of legs and antennae. Registration No. CNU-C-LB2010813, housed in Key Lab of Insect Evolution & Environmental Change, Capital Normal University, Beijing, China.

#### Horizon and locality

Daohugou Village, Shantou Township, Ningcheng County, Inner Mongolia, China; Jiulongshan Formation, the Middle Jurassic (Bathonian- Callovian boundary).

#### Description

Body small, flattened, densely covered with tubercles (Figure 5C).

*Head capsule:* slightly longer than wide, but appearing elongated; distinctly narrower anteriorly than at ocular region; distinctly prognathous; without macroscopic tubercles on dorsal surface; anterior margin shallowly concave; temples distinctly shorter than eyes, moderately projecting laterally. Compound eyes posterior, approximately round in outline, distinctly convex and protruding laterally; neck region moderately constricted, with conspicuous postocular extensions (Figure 4A).

*Head appendages:* Antennae inserted laterally in front of eyes; filiform, thin, with 9 visible antennomeres; short, not reaching posterior margin of prothorax; scape subtriangular, longer than wide, wider apically than basally; pedicel thin, shorter than scape; antennomere 3 longer than others, nearly as long as pedicel and antennomere 4 combined; other antennomeres nearly equal in length, slightly widening distally; apical antennomere parallel-sided. Mandibles large, without recognizable teeth along horizontal mesal surface (Figure 4A). Remaining elements of ventral mouthparts not recognizable.

*Pronotum:* rounded at sides, widest posterior to midlength and not explanate, slightly wider than base of head; about 4/5 times as long as wide at anterior margin; posterior edge about as wide as pronotal length; anterior and posterior pronotal edges almost straight; central disc flattened, without elevation but with large tubercles (Figure 4A).

*Scutellar shield:* exposed, roughly triangular.

*Elytra:* maximum width at elytral midlength; individual elytron 3.7 times as long as wide; epipleural rim rather narrow; elytral disc enclosed by dorsolateral ridges, also flat or slightly concave except for anteriormost part; dorsolateral ridges anteriorly connected with elytral shoulders; elytra reaching slightly beyond abdominal apex; appearing dehiscent in posterior fourth (possibly an artifact of compression during the fossilization) (Figure 4A).

*Thoracic venter:* anapleural cleft converging posteriorly towards procoxae; prothoracic notopleural and sternopleural sutures enclose triangular, anteriorly narrowing pleura, which meet the notopleural suture at a narrow point; protrochantin not recognizable. Longitudinal suture (discrimen) present on mesoventrite. Metaventrite trapezoidal, distinctly narrowing anteriorly; posterior margin 2.1 times as wide as length at midline; anterior margin distinctly narrower than posterior margin, about as wide as mesocoxae combined; mesocoxae medially adjacent, separated by short, triangular, apically pointed process of metaventrite; metathoracic discrimen invisible; metakatepisternal suture present; metatrochantin present and exposed between hind margin of katepisternum and coxa (Figure 4B). Individual structures of metapleuron not recognizable; metanepisternum apparently broad anteriorly and narrowing towards end of segment.

*Legs:* procoxae nearly spherical, protruding, medially adjacent; protrochanter small, approximately triangular; profemur long, narrowing distally, with strongly convex posterior margin, distinctly thicker than protibia and slightly longer; protibia short, slender, slightly widening distally. Mesocoxae nearly triangular, larger than procoxae, medially adjacent; mesotrochanter small, longer than wide; mesofemora parallel-sided, with very distinct anterior and posterior margins, proximally wider than basally, distinctly thicker than mesotibia and equally long; mesotibia subparallel, with a short spur on the internal surface; mesotarsus with 4 visible tarsomeres; tarsomere 1 elongate, longer than others, tarsomeres 2–4 short. Metacoxae transverse, distinctly reaching beyond lateral margin of metaventrite, triangular, without metacoxal plates, posteromesal apices rounded, extending over mid-length of first visible abdominal ventrite; metatrochanter medium-sized, longer than wide; metafemora reaching beyond lateral margin of body; metatibia almost parallel-sided; metatarsi with 2 visible tarsomeres; tarsomere 1 elongate, longer than tarsomere 2.

*Abdomen:* narrowing towards apex from the base of ventrite 4; first ventrite longer than other ones; last ventrite (=sternite VII) 1.4 times as long as the previous one; apex rounded.

Dimensions (mm): body length 9.0, width 3.0, head length 1.5, width 1.6, anterior edge of pronotum 1.2, posterior edge of pronotum 1.7, pronotal width 1.5, elytral length 5.9, width 1.6.

#### Comments

The new species is similar to *Omma antennatum* Ponomarenko, 1997, *O. avus* Ponomarenko, 1969 and *O. rutherfordi* Lawrence, 1999 in the body size. It differs from *O. avus* Ponomarenko, 1969 by elytra almost completely lacking veins and cells, and the laterally projecting temples. It differs from *O. antennatum* Ponomarenko, 1997 in the following features: profemora not inflated anteriorly and posteriorly, pronotal angles completely rounded, not prominent; in contrast to *O. rutherfordii* Lawrence, 1999 the head behind the eyes is wide and not abruptly narrowed.

Tribe **Tetraphalerini Crowson, 1962**

Genus ***Tetraphalerus*** Waterhouse, 1901

*Tetraphalerus* Waterhouse, 1901, Ann. Mag. Nat. Hist. Ser 7, Vol. Vii:520–523; Heller, 1913, Wien. Ent. Zeit. 32: 235; Bruch, 1925, Physis. 7: 201–204; Monrós and Monrós, 1952, An. Soc. Cient. Argent. 154: 23–39; Crowson, 1962, Ann. Mag. Nat. Hist. 13(5): 147–157; Atkins, 1963, Can. Ent. 95: 140–162; Ponomarenko, 1964, Paleontol. Jour. 4: 45–55; Ponomarenko, 1966, Entomolog. Oboer. 1: 138–143; Ponomarenko, 1969, Tru. Paleontol. Instit. A. N. SSSR, 125: 70–115; Lin, 1976, Acta Palaeontol. Sin. 15(1): 97–115; Ponomarenko, 1986, Trans. Joint Soviet-Mongolia Paleon. Exped. 28, 84–107; Ponomarenko, 1993, Tru. Paleontol. Instit. A. N. SSSR, 252: 17–20; Ren *et al.*, 1995, Seism. Pub. H. pp77-78; Ponomarenko, 1997, Paleontol. Jour. 31(4): 389–399; Ponomarenko, 2000, Acta Geolog. Hispan. 35, n^o^1-2: 47–52; Sorinao and Delclòs, 2006, Acta Palaeontol. Pol. 51(1): 185–200; Tan, Ren and Shih, 2007, Ann. Zoo. (Warszawa), 57(2): 231–247.

#### Type species

*Tetraphalerus wagneri* Waterhouse, 1901; Recent.

#### Included species

25 species have been known before this study. *T. wagneri* Waterhouse, 1901 and *T. bruchi* Heller, 1913 are two extant species from South America. The others are all fossil species from the Mesozoic of Siberia, Central Asia, Western Europe, Western Australia and China. In addition, *T. decorosus* sp. nov. is described below.

#### Emended diagnosis

Head narrow anteriorly and broad in post-ocular region, with ridges and lobes. Antennae filiform or somewhat moniliform, as long as or slightly shorter than head and prothorax taken together; longitudinal, distinct antennal groove present on ventral side of head; antennomere 3 slightly long than antennomere 4. Clypeus and frons fused; labrum short, transverse. Mandibles prominent, incurved; temples as long as or longer than eyes. Pronotum nearly rectangular, as wide as head or narrower, with lateral longitudinal line; central part of pronotum strongly raised; procoxal cavities contiguous. Elytra 1.5 times to twice as wide as prothorax. Legs short, mesofemora not extending well beyond side margins of body; tarsal segment 4 simple. Abdominal sternites arranged in one plane, abutting, not overlapping.

#### Note

The emended diagnosis is based on the type species and the new material.

### ***Tetraphalerus decorosus*** sp. nov. 

#### Diagnosis

Head slightly longer than wide; antennae moniliform; antennomeres 5–10 short, distinctly broader than antennomeres 3–4, distally widening; apical antennomere appearing inflated. Maximum width of elytra posterior to midlength; abdominal ventrites narrowing towards apex from the base of ventrite 3; first abdominal ventrite longer than the other ones; last ventrite (=sternite VII) 1.3 times as long as the previous one; hind margin evenly rounded.

#### Etymology

Name derived from the Latin word ‘*decorosus*’, *-a, -um*, referring to the new species preserved completely.

#### Type Material

Holotype: a nearly complete adult with body, elytra, and parts of legs and antennae. Registration No. CNU-C-LB2010812, housed in Key Lab of Insect Evolution & Environmental Change, Capital Normal University, Beijing, China.

#### Horizon and locality

Daohugou Village, Shantou Township, Ningcheng County, Inner Mongolia, China; Jiulongshan Formation, the Middle Jurassic (Bathonian- Callovian boundary).

#### Description

Body small, surface densely covered with large tubercles (Figure 5D).

*Head capsule:* with distinctly protruding compound eyes at middle region of head approximately semicircular in outline, slightly wider than long; distinctly narrower anteriorly and posteriorly than at ocular region and temples; distinctly prognathous; anterior clypeal margin straight; frontoclypeal transverse line not recognizable; temples distinctly shorter than eyes, moderately projecting laterally. Ocelli absent. Two distinct circular, flat macro-tubercles present between posterior margins of eyes; median epicranial suture indistinctly visible; neck region distinctly constricted, as long as compound eyes (Figure 2A). Antennal groove on ventral side of head not visible.

*Head appendages:* Antennae inserted laterally in front of the eyes; moniliform, short, not reaching posterior margin of prothorax, wider apically than basally; scape approximately triangular, widening distally; pedicel small; antennomere 3 as long as pedicel and antennomere 4 combined. Mandibles exceptionally long and distinctly protruding; without recognizable tooth along horizontal mesal surface; outer margin curved; neck region moderately constricted, approximately as long as compound eyes (Figure 2A). Two maxillary palpomeres visible; remaining elements of ventral mouthparts not recognizable.

*Pronotum:* transverse, slightly wider than head; about 1.1 times as wide as anterior edge; about 1.3 times as wide as posterior edge; anterior and posterior margin almost straight; anterior and posterior angles of pronotum not prominent; lateral margin moderately serrate and moderately explanate; prothoracic hypomeron narrow; central disc near lateral pronotal carinae bearing two oblong flat convexities (Figure 2A).

*Scutellar shield:* exposed, shield-like, distinctly longer than wide; lateral margins concave, slightly widening anteriorly.

*Elytra:* individual elytron about 3.2 times as long as wide; pronounced dorsolateral ridges and sharply pronounced lateral edge enclose flat or even slightly concave epipleural rim; elytral disc enclosed by dorsolateral ridges, also flat or slightly concave except for anteriormost part; epipleural rim slightly widening in anterior 1/3, bearing one row of cells, narrowing towards apex; dorsolateral ridges anteriorly connected with elytral shoulders (Figure 2A); longitudinal ridges with small tubercles, with 9 rows of cells; elytral cells (=window punctures) moderately distinct, small, polygonal, with 4 black maculae on their margins; about 28 cells arranged in a row; regular rows of large cells along lateral elytral margin absent (Figure 2C); elytra reaching slightly beyond abdominal apex; appearing dehiscent in posterior third (possibly an artifact of compression during fossilization).

*Thoracic venter:* Individual structures of meso- and metaventrites not recognizable; metakatepisternal suture present; metatrochantins present and exposed between hind margin of katepisternum and coxa (Figure 2B).

*Legs:* procoxae medially adjacent, medium-sized, cone-shaped but rounded apically; protrochanter medium sized, longer than wide; profemur long, with strongly convex posterior margin; distinctly thicker than proximally curved and slender protibia and slightly longer; protibia short, slightly widening distally; protarsus 5-segmented; tarsomeres 2–4 short, only slightly wider than long; tarsomere 5 longer than others, with one claw; mesocoxae inconspicuous, slightly smaller than procoxae, apparently globular in outline, separated from each other; metacoxae transverse, triangular; without metacoxal plates; posteromesal apices rounded, far from posterior margin of first abdominal ventrite (Figure 2B).

*Abdomen:* narrowing towards apex from the base of ventrite 3; hind margin evenly rounded (Figure 2B).

Dimensions (mm): body length 8.7, width 3.1, head length 1.6, width 1.4, anterior edge of pronotum 1.6, posterior edge of pronotum 2.0, pronotal width 1.5, elytral length 5.5, width 1.7.

#### Comments

The new fossil specimen is assigned to *Tetraphalerus* based on the following suite of characters: head nearly as wide as pronotum, antennae short, third antennomere longer than fourth, and the pronotum with lateral pronotal carinae. The presence of two prominences on the vertex suggests a placement of the new species to the *T. bruchi* series. The new species is distinctly different from other species in several features: head wider than long; antennae moniliform.

### Phylogenetic results

(a) *Tree for fossil and extant Ommatidae*

**Figure 6:**
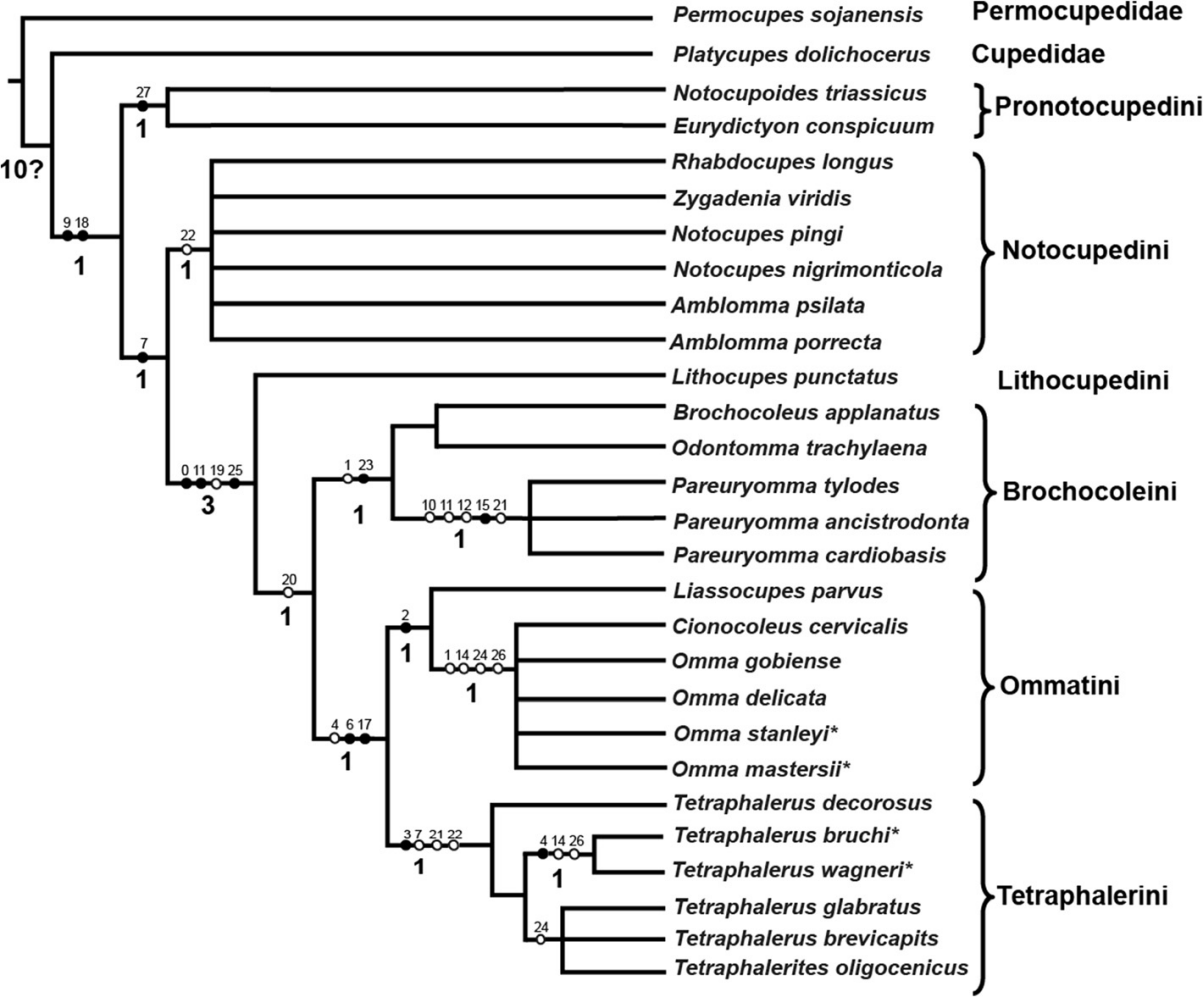
**Results of the phylogenetic analyses as represented by strict consensus tree by NONA.** Black circles indicate non-homoplasious changes; white circles indicate homoplasious characters; numbers above the branches of the strict consensus tree indicate character numbers; Bremer support values presented above the branches.

Nine most maximum parsimonious trees were obtained in the NONA analysis (tree length = 57 steps, CI = 0.50, RI = 0.80). A similar result was obtained in analyses using TNT (9 trees, 57 steps). The strict consensus tree obtained with NONA with Bremer support values mapped on branches is shown in Figure 6. The cladistic result obtained by TNT is similar to the results by NONA (see Additional file 2). The constrained analysis forcing the monophyly of *Tetraphalerus* + *Odontomma* and *Pareuryomma* + *Notocupes* yielded 434 most-parsimonious trees (tree length = 67 steps, CI = 0.42, RI = 0.76; see Additional file 3: Figure S1B), with 10 additional steps compared to the unconstrained results. The constrained analysis forcing the monophyly of *Tetraphalerus* and *Odontomma* yielded 972 most-parsimonious trees (tree length = 61 steps, CI = 0.48, RI = 0.80; see Additional file 3: Figure S1C). The constrained analysis forcing the monophyly of *Pareuryomma* + *Notocupes* yielded 140 most-parsimonious trees (tree length = 63 steps, CI = 0.46, RI = 0.79; see Additional file 3: Figure S1D)

The constrained analysis forcing the monophyly of *Tetraphalerus* + *Odontomma* and *Pareuryomma* + *Notocupes* yielded 434 most-parsimonious trees (tree length = 67 steps, CI = 0.42, RI = 0.76; see Additional file 3: Figure S1), which need nine additional steps more than the most parsimonious results. The constrained analysis forcing the monophyly of *Tetraphalerus* and *Odontomma* yielded 972 most-parsimonious trees (tree length = 61 steps, CI = 0.48, RI = 0.80; see Additional file 3: Figure S1). The constrained analysis forcing the monophyly of *Pareuryomma* + *Notocupes* yielded 140 most-parsimonious trees (tree length = 63 steps, CI = 0.46, RI = 0.79; see Additional file 3: Figure S1).

(b) Apomorphies of selected clades

The monophyly of Ommatidae is supported by two apomorphic characters: the antenna not reaching hind margin of prothorax posteriorly (ch. 9), and the medially adjacent procoxae (ch. 18). In all trees, *Notocupoides* and *Eurydictyon* are a monophyletic group (sharing ch. 27) and form the sister group of all other included ommatid taxa. Ommatidae excl. *Notocupoides* + *Eurydictyon* is supported by a ratio labral width/clypeal width less than 1 (ch. 7:1).

Ommatidae excluding the traditional tribe Notocupedini (including the genera *Notocupoides*, *Eurydictyon*, *Notocupes*, *Amblomma*, *Rhabdocupes*, and *Zygadenia*) are monophyletic. This clade is supported by the absence of a longitudinal bulge or keel on the dorsal head surface (ch. 0:1), the distinct elongation of the third antennomere (ch. 11:1), and the small size of the window punctures on the elytron (ch. 25:1).

A clade including the genera *Brochocoleus*, *Odontomma*, *Pareuryomma*, *Liassocupes*, *Omma*, *Cionocoleus*, *Tetraphalerus* and *Tetraphalerites* is suggested by abutting abdominal sterna (ch. 20:0). However, this condition is arguably plesiomorphic. *Lithocupe*s, the only single genus of the tribe Lithocupedini, is placed as the sister taxon of this clade in all trees, shared derived features are the above mentioned apomorphies of Ommatidae excl. the traditional Notocupedini.

The three genera *Brochocoleus*, *Odontomma* and *Pareuryomma* constitute a clade defined by one non-homoplasious character, an epipleural rim containing more than one row of cells near its basal part (ch. 23:1), and one homoplasious character, the absence of the posteromesal dorsal protuberance of the head (ch. 1:1). *Liassocupes*, *Omma* and *Cionocoleus* are supported as a clade by the absence of the median epicranial (=coronal) suture on the dorsal head (ch. 2:1). The two genera, *Tetraphalerus* and *Tetraphalerites* share a non-homoplasious apomorphic character state, the presence of ventrolateral antennal grooves (ch. 3:1). However, this ventral groove is not visible in the fossils of *Tetraphalerus*. There are several homoplasious characters also supporting this group, such as a strongly transverse labrum (as wide as the clypeus) (ch. 7:0) and a mesofemur not extending beyond side margins of body (ch. 21:1). The two lineages are likely sister taxa, as suggested by the non-homoplasious absence of the frontoclypeal suture (ch. 6:1), which reduced countless times in Coleoptera, and the narrower lateral pronotal projection (ventrally corresponding with pronotal epipleura) (ch. 17:1).

## Discussion

### (a) *Systematics of* Ommatidae

The interrelationships within the family Ommatidae are still uncertain. Conventionally, Ommatidae is divided into four subfamilies: Lithocupedinae, Notocupedinae, Ommatinae and Tetraphalerinae [7]. Herein, we conducted a cladistic analysis to infer the phylogenetic relationships of the extinct and extant genera within the family. Although the generic relationships are still not well resolved, the results of the phylogenetic analysis suggest six monophyletic groups: Pronotocupedini, Notocupedini, Lithocupedini, Brochocoleini, Ommatini and Tetraphalerini. Only the two subfamilies Lithocupedinae and Tetraphalerinae were recovered, while the other two, Notocupedinae and Ommatinae, turned out as paraphyletic. Therefore, we propose to use these six tribes to replace the traditional four subfamilies [7] in presenting the higher-level relationships within Ommatidae.

The paraphyly of the traditional of Notocupedinae [7], including *Notocupoides*, *Eurydictyon*, *Rhabdocupes*, *Zygadenia* (*Notocupes*), and *Amblomma*, implies a reassessment of taxa previously assigned to this subfamily. The monophyletic tribe Notocupedini apparently includes only *Rhabdocupes*, *Zygadenia* (*Notocupes*), and *Amblomma*. The placement of the Lower Triassic genera *Notocupoides* and *Eurydictyon* as the sister group of the remaining Ommatidae is tentatively supported in the parsimony analysis. Thus, these two genera cannot be included in the tribe Notocupedini. These two ancestral genera, the earliest representatives of Ommatidae in the fossil record, are assigned to a new tribe Pronotocupedini, as the sister group of all other ommatids.

Ponomarenko [23] considered *Notocupes* Ponomarenko, 1964 as a junior subjective synonym of *Zygadenia* Handlirsch, 1909. However, in a more recent study [24,25], he pointed out that the *Notocupes* was described based on the study of more or less complete bodies of the beetle, while the genus *Zygadenia* was proposed only based on the elytra. Therefore, Ponomarenko [24] concluded that the synonymy of *Notocupes* under *Zygadenia* would impede the placement of the genus, and *Zygadenia* should be regarded as an informal morphogenus. The results of the present analyses, as shown in Figure 6, also support this view.

The relationships of the genera *Amblomma*, *Notocupes* and *Rhabdocupes* are still unclear. These genera are characterized by a peculiar venation of their elytra with double rows of cells between the veins and only 2 sutural main veins meeting before elytral apex. *Notocupes*, which was the most common and diverse genus among Mesozoic ommatids, is recorded from the Early Triassic to the Early Paleocene [3,20,23,24]. Except for the difference in the proportions between the pedicel and third antennomere, the species of the Early Cretaceous genus *Amblomma* and the Early Triassic genus *Rhabdocupes* could be members of *Notocupes* Ponomarenko, 1964 [16]. However, this requires a careful re-examination of specimens assigned to these genera.

The subfamily Ommatinae turned out as a paraphyletic assemblage, and was divided into two separate monophyletic groups, Brochocoleini and Ommatini in our analyses. Three genera *Brochocoleus*, *Odontomma*, and *Pareuryomma* were attributed to the tribe Brochocoleini. This tribe was first described by Hong [43] as a new archostematan family Brochocoleidae. After Hong’s work, Ponomarenko [44] also found some complete ommatid beetles with wide elytral epipleural rims with several rows of cells from the Jurassic of Mongolia. However, after studying these fossils, Ponomarenko concluded that the placement of Brochocoleidae was unjustified, as it was based on a single elytral fossil. He proposed a status as a tribe Brochocoleini within Ommatinae. In our analysis, Brochocoleini is the sister group of Tetraphalerini and Ommatini. A presumably synapomorphic feature of the tribe is the presence of more than one row of cells near the basal part of the elytral epipleural rim (see above). Based on this feature [22], *Brochocoleus aphaleratus* (Ponomarenko 1969) [3] was transferred from the genus *Tetraphalerus* to *Brochocoleus* by Ponomarenko in 1997.

The genus *Odontomma* was erected by Tan, Ren *et* Ge, 2006 which is characterized by a flattened dorsal head surface and a wider epipleural rim. Kirejtshuk *et al.*[41] considered it should be a synonym of *Tetraphalerus* Waterhouse, 1901. In our cladistic analysis, *Odontomma* was assembled to Brochocoleini instead of showing close relation to *Tetraphalerus.* In the topological constraint analysis, enforcing the monophyly of *Tetraphalerus* + *Odontomma* gained more steps than the unconstrained analysis, implying *Odontomma* should be a valid genus.

The other genus *Pareuryomma* was assigned to Brochocoleini, which is incompatible with Kirejtshuk’s assumption as a synonym of the genus *Notocupes* (*Zygadenia*) Ponomarenko, 1964 [41]. The constrained analysis forcing the monophyly of *Pareuryomma* and *Notocupes* gained four additional steps comparing with the unconstrained results. In fact, *Pareuryomma* differs from the *Notocupes* (*Zygadenia*) in several characters: the absence of protuberances or longitudinal bulges or keels on the dorsal surface of the head, moniliform antennae, a quadrate pedicellum, a wide epipleural rim, and the abutting arrangement of the abdominal sterna. Therefore, we consider the arrangement of *Pareuryomma* within Brochocoleini is more suitable.

Three genera *Liassocupes*, *Cionocoleus* and *Omma* are placed in a subgroup of the tribe Ommatini, which is supported by the following characters: the absence of the median epicranial suture on the head, the absence of the elevation on the pronotal disc, and a propleuron indistinctly fused to the sternum.

The monophyly of Tetraphalerini was weakly supported in our analyses by one character not identifiable in fossils. The systematic position of the genus *Tetraphalerites* Crowson, 1962 remains rather unclear. This genus includes only one species, *Tetraphalerites oligocenicus* Crowson, 1962. Ponomarenko transferred *Tetraphalerites* to the tribe Brochocoleini in 2006 [24]. The visible ventral structure (including the impressions of middle and hind femora) are very similar like that to recent *Tetraphalerus*[2], but the elyra are not quite like those of this genus. Because of the poor preservation of the specimens with many characters not recognizable (e.g., antennal groove), we tentatively attributed *Tetraphalerites* to Tetraphalerini based on a single recognizable apomorphy, which is a mesofemur not extending beyond side margins of body.

### (b) *Evolutionary trends and origin of* Ommatidae

The recent phylogenetic results shed light on the evolutionary trends of key characters (Table 2) and evolutionary processes.

**Table 2:** Evolutionary trends of key characters for Ommatidae

	**Characters**	**Pronotocupedini**	**Notocupedini**	**Lithocupedini**	**Brochocoleini**	**Ommatini**	**Tetraphalerini**
1	Pentagonal enlarged window punctures along elytral suture	present	absent	absent	absent	absent	absent
2	Ratio of length of 3^rd^ and 4^th^ segments of antenna	equal in length	equal in length	3^rd^ longer than 4^th^ antennomere	3^rd^ longer than 4^th^ antennomere	3^rd^ longer than 4^th^ antennomere	3^rd^ longer than 4^th^ antennomere
3	Size of window punctures enlarged, separated by very distinct rib-like ridges	present	present	absent	absent	absent	absent
4	Abdominal ventrites	overlapping on fore border of next	overlapping on fore border of next	overlapping on fore border of next	flat	flat	flat
5	Gular sutures	complete	complete	complete	incomplete	incomplete	absent
6	Epipleural rim	one row of cells	one row of cells	one row of cells	more than one row of cells	one row of cells	one row of cells
7	Median epicranial suture on the dorsal head surface	present	present	present	present	absent	present
8	The ventral head surface with lateral antennal grooves	absent	absent	absent	absent	absent	present

Pentagonal enlarged window punctures along the elytral suture is an unusual autapomorphy of the tribe Pronotocupedini. The species of Notocupedini have the 3^rd^ and 4^th^ antennomeres equal in length, the gula not converging posteriorly, and the size of window punctures enlarged and separated by very distinct rib-like ridges. The gula of Lithocupedini is similar to that of Notocupedini, but the third antennomere is longer than the fourth one, the rows of punctures are of normal size. The epipleural space of the elytron in Brochocoleini is very wide with more than one row of proximal cells. Except for this archostematan tribe, only two families have a wide epipleural margin with several rows of cells: Tshekardocoleidae in the Permian and Labradorocoleidae in the Early Cretaceous. After the Permian, the wide epipleural rim apparently evolved for a second time in Brochocoleini and then another time in Labradorocoleidae in the Cretaceous. Ommatini have gular sutures that are incomplete, not reaching the hind margin of the head capsule, and the median epicranial suture on the dorsal head surface is absent. Moreover, the gular sutures in Tetraphalerini species are absent. Lateral antennal grooves are present on the ventral side of the head in species of Tetraphalerini, which is an autapomorphy of this group.

**Figure 7:**
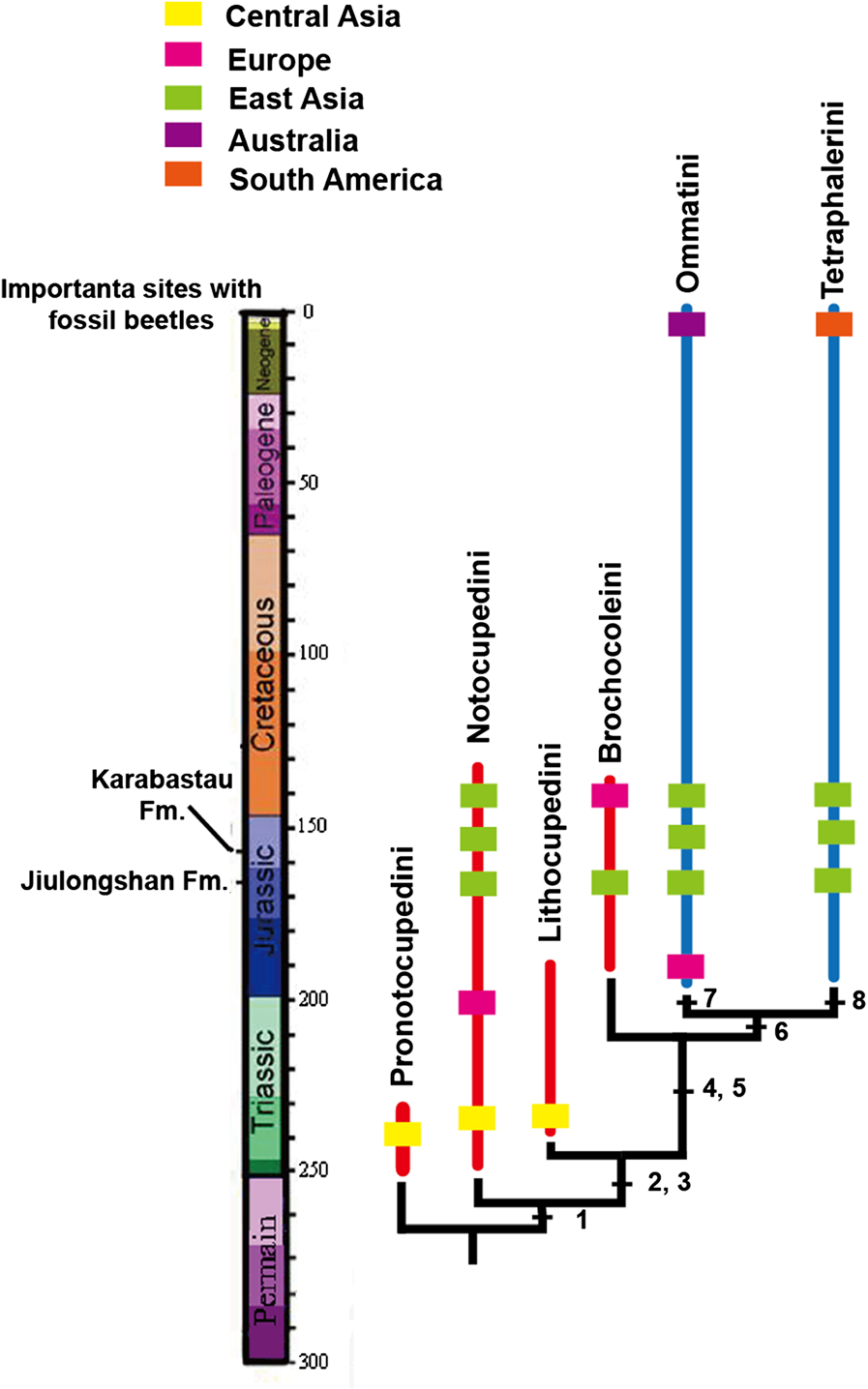
**Phylogenetic relationships among major lineages of Ommatidae.** Red lines are the known extinct taxa, blue lines are extant taxa. Different color dots show different sites with fossils. Significant characters in Ommatidae phylogeny: 1. Pentagonal enlarged window punctures along elytral suture; 2. Third antennomere distinctly elongated; 3. Regular row of large window punctures present along external elytral margin; 4. Abdominal ventrites flattened; 5. Gular sutures incomplete or absent; 6. Epipleural rim with more than one row of cells; 7. Median epicranial suture on the dorsal head surface; 8. Head with ventrolateral antennal grooves.

The species of the tribe Pronotocupedini is consistent with the early appearance of the group in the ommatid fossil record. The evolutionary innovation linked with the earliest splitting event in the Lower Permian is a shortening of the elytra, which then fit better with the shape of the abdomen [12], distinct modifications of the elytral venation [45], and a distinctly narrowed prosternal process [45]. The tribe Notocupedini was present from the Early Triassic of Kyrgyzstan to the Early Cretaceous of Russia and Spain [3]. The tribe Lithocupedini was represented in Central Asia from the Early Triassic to the Early Jurassic. The more advanced Ommatini appeared in the Lastest Triassic [2,3] and became abundant in Europe and Asia during the Mesozoic. However, only four extant species of *Omma* occur in Austrialia today. The new fossil species of *Omma* described here is the first Chinese representative of this genus, thus, extends its geographical distribution from Central to Eastern Asia. The earliest occurrence of Tetraphalerini is in the Lower Jurassic of Kyrgyzstan [3]. Today only two species of *Tetraphalerus* have been reported from South America [6,8]. The phylogenetic relationships of the genera of Ommatidae are summarized in Figure 7.

## Conclusions

The new discoveries of ommatid fossils from the Mesozoic of China provided insights into morphological changes, the phylogeny, and the evolution of Ommatidae. Based on the “International Code of Zoological Nomenclature (fourth edition)”, we amend the genus name from “*Euryomma*” to “*Pareuryomma*”, in order to differentiate the fossil beetles found in China from the flies of the family Fanniidae. Based on our phylogenetic analysis, Ommatidae was divided into six monophyletic tribes: Pronotocupedini, Notocupedini, Lithocupedini, Brochocoleini, Ommatini and Tetraphalerini, instead of the traditional four subfamilies. Meanwhile, the phylogenetic analysis showed that the genera *Pareuryomma* and *Odontomma* should be assigned to the tribe Brochocoleini. They were distinctly different from the genera *Notocupes* (*Zygadenia*) and *Tetraphalerus*, respectively. Our phylogenetic results shed light on the possible evolutionary processes in Ommatidae: Pronotocupedini appeared in Kirghiz no later than 250 Mya ago, followed by the rapid evolution of ommatids across Pangaea, until the break-up of Pangaea after the Middle Jurassic. They split into six tribes not later than in the Early Jurassic.

## Authors’ contributions

TJJ carried out the fossil processing, photography, figure preparation, data analysis and interpretation, manuscript drafting and finalization. DR did the fieldwork, collected the specimens, and manuscript modification. WYJ and YXK participated in the data analysis and manuscript modification.

## Supplementary Material

Additional file 1 **Character matrix for fossil and extant taxa.** “?": Character missing. “-”: Character inapplicable. Click here for file

Additional file 2 Nine most parsimonious trees by TNT. Click here for file

Additional file 3 **Figure S1.** Results of the cladistic analysis. A. The consensus tree from unconstrained analysis. B. The constrained consensus tree enforcing *Tetraphalerus* + *Odontomma* and *Pareuryomma* + *Notocupes*. C. The constrained consensus tree enforcing *Tetraphalerus* + *Odontomma*. D. The constrained consensus tree enforcing *Pareuryomma* + *Notocupe*. Click here for file
