# Supplementary material for: New fossil species of ommatids (Coleoptera: Archostemata) from the Middle Mesozoic of China illuminating the phylogeny of Ommatidae
Source: BMC Evol Biol. 2012 Jul 9;12:113. doi: 10.1186/1471-2148-12-113 (PMC3518168; doi:10.1186/1471-2148-12-113)
Supplement: Additional file 3 — Figure S1. Results of the cladistic analysis. A. The consensus tree from unconstrained analysis. B. The constrained consensus tree enforcing Tetraphalerus + Odontomma and Pareuryomma + Notocupes. C. The constrained consensus tree enforcing Tetraphalerus + Odontomma. D. The constrained consensus tree enforcing Pareuryomma + Notocupe. [file 1471-2148-12-113-S3.docx]

Additional file 3. Results of the constrained analysis.





A. Strict consensus tree from unconstrained anlaysis. B. Strict consensus tree of constrained analysis forcing the monophyly of *Tetraphalerus* + *Odontomma* and *Pareuryomma* + *Notocupes*. C. Strict consensus tree of constrained analysis forcing the monophyly of *Tetraphalerus* and *Odontomma*. D. Strict consensus tree of constrained analysis forcing the monophyly of *Pareuryomma* and *Notocupes*.
